# Exploiting Cell Death Pathways for Inducible Cell Elimination to Modulate Graft-versus-Host-Disease

**DOI:** 10.3390/biomedicines5020030

**Published:** 2017-06-14

**Authors:** Corey Falcon, Mustafa AL-Obaidi, Antonio Di Stasi

**Affiliations:** Department of Medicine, Hematology-Oncology, Stem Cell Transplantation and Cellular Therapies, The University of Alabama at Birmingham, Birmingham, AL 35294, USA; coreyfalcon@uabmc.edu (C.F.); mobaidi@uab.edu (M.A.-O.)

**Keywords:** cellular therapies, graft versus host disease (GVHD), inducible suicide genes, apoptosis, necroptosis

## Abstract

Hematopoietic stem cell transplantation is a potent form of immunotherapy, potentially life-saving for many malignant hematologic diseases. However, donor lymphocytes infused with the graft while exerting a graft versus malignancy effect can also cause potentially fatal graft versus host disease (GVHD). Our group has previously validated the inducible caspase-9 suicide gene in the haploidentical stem cell transplant setting, which proved successful in reversing signs and symptoms of GVHD within hours, using a non-therapeutic dimerizing agent. Cellular death pathways such as apoptosis and necroptosis are important processes in maintaining healthy cellular homeostasis within the human body. Here, we review two of the most widely investigated cell death pathways active in T-cells (apoptosis and necroptosis), as well as the emerging strategies that can be exploited for the safety of T-cell therapies. Furthermore, such strategies could be exploited for the safety of other cellular therapeutics as well.

## 1. Introduction

Effector T-cells are key players of adaptive cellular immune responses, protecting the host from infectious pathogens, cancer, and other cells foreign to the body. In the context of allogeneic hematopoietic stem cell transplantation (allo-HSCT), engrafted T-cells of donor origin also contribute to the so-called graft-versus-tumor effect, which together with the conditioning regimen, aids in tumor surveillance and/or eradication [1]. However, T-cells can also trigger potentially fatal graft-versus-host-disease (GVHD) by attacking normal host tissues, and, therefore, immune-suppressive treatments are routinely adopted after allo-HSCT. As a matter of fact, patients receiving transplants from HLA matched donors receive immune-suppressive agents both during the peri-transplant period and for about six months after transplant. Transplants from haploidentical donors, characterized by increased risk of GVHD due to high HLA disparity, have been performed with in vitro or in vivo T-cell depletion strategies [2]. Although T-cell graft manipulation can be effective in reducing the proportion and/or function of alloreactive T-cells, alloreactivity is not completely eliminated and the risk of GVHD remains. More recently, post-transplant cyclophosphamide has been widely employed after haploidentical HSCT [3], enabling the infusion of T-cell-replete grafts with acceptable incidence and severity of GVHD. However, since once GVHD develops the elimination of the infused T-cells by pharmacological means is generally slow and incomplete, the ability to conditionally eliminate T-cells when required is an appealing approach. One strategy, more widely investigated in the haploidentical setting, involves gene modification of donor T-cells with a safety switch, allowing for on demand elimination of the infused cells in case of adverse events, such as GVHD [4]. Two suicide genes have been widely investigated in the clinical setting, the herpes-simplex-thymidine-kinase (*HSV-TK*) suicide gene [5] and the inducible caspase-9 (*ΔiC9*) suicide gene, the latter validated by our group [6,7,8]. While HSV-TK phosphorylates nucleoside analogs, such as ganciclovir (GCV), and the resulting triphosphate form incorporates into DNA via the action of DNA polymerase leading to chain termination and cell death [9], the *ΔiC9* suicide gene activates the apoptotic pathway in gene modified cells after administration of an otherwise inert agent inducible of dimerization [10]. Importantly, in our experience, the infusion of ΔiC9 donor lymphocytes avoided the use of immunosuppressive therapy [6,7,8], with potentially less off-target effects on immune response and eventual organ damage.

This review will summarize the molecular pathways of programmed cell death and the in vitro and in vivo investigations of suicide gene strategies for the safety of T-cell therapies with a focus on prevention/treatment of GVHD. Such strategies could be exported to other T-cell therapies as well as to other cellular therapeutics.

## 2. Pathways of Programmed Cell Death in T-Cells

T-cell survival is influenced by the signals the cell receives through the (i) T-cell receptor (TCR), co-stimulatory molecules, including CD28; (ii) adhesion molecules; (iii) cytokines and (iv) other pro- or anti-apoptotic molecules. All of these factors are important for the optimal function of T-cells. In fact, in the absence of appropriate survival signals, T-cells undergo activated cell-autonomous death (ACAD) [11], whereas TCR restimulation of already expanded T-cells in the absence of appropriate co-stimulation signals leads to activation-induced cell death (AICD) [12]. Homeostasis of T-cells during T-cell development and antigen specific responses, important to avoid normal organ damage and lymphoproliferation, is maintained through activation of programmed cell death pathways, most notably, apoptosis [13,14]. Interestingly, after clonal expansion, a subset of memory T-cells that are resistant to death by apoptosis [15] remain to protect for future rechallenges [15]. Caspases exert a regulatory and/or executioner role in apoptosis [16,17]. Caspases are produced as catalytically inactive zymogens and undergo proteolytic processing during activation [18]. The effector caspases are activated by initiator caspases, which themselves must first be activated. All initiator caspases are composed of the death domain (DD) (80–100 amino acids).

**Figure 1:**
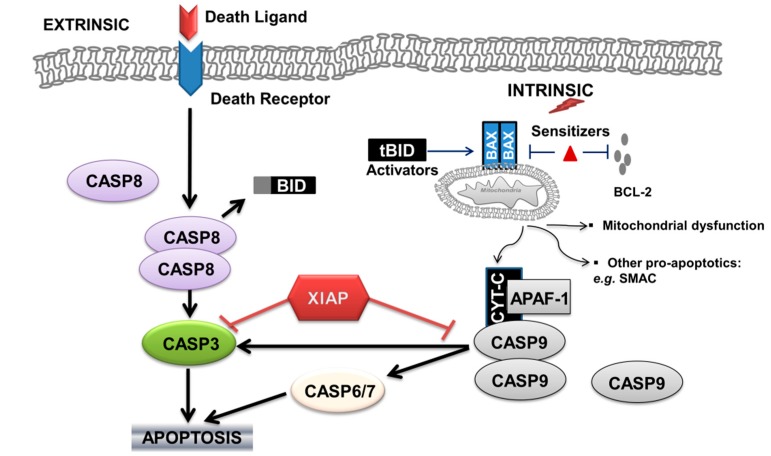
Schematic representation of the extrinsic and intrinsic apoptotic pathway in T-lymphocytes. Arrows indicate activation, red and black T bars indicate inhibition. CASP: caspase; BID: BH3-interacting-domain death agonist; BAX: BCL-2-associated X protein; BCL-2: B cell lymphoma 2; XIAP: X-linked inhibitor of apoptosis protein; CYT-C: cytochrome C; APAF-1: apoptotic protease-activating factor 1; SMAC: second mitochondria-derived activator of caspases.

Caspase-dependent apoptosis can be activated through the extrinsic cell-death-receptors pathway, and/or through the intrinsic mitochondrial pathway (Figure 1).

In the extrinsic apoptotic pathway, cell-death-receptor-adaptor molecules (death-inducing signaling complex (DISC)) deliver pro-apoptotic signals [19] that are transmitted by ligands [20] such as tumor- necrosis factor (TNF), CD95 ligand/FAS ligand (CD95L/FASL) and TNF-related apoptosis-inducing ligand (TRAIL) after binding to the respective death receptor. For example, stimulation of tumor necrosis factor receptor 1 (TNFR1) by TNF recruits TNFR1-associated death domain (TRADD), with formation of the TRADD-dependent complex IIa (FAS-associated death domain (FADD), pro-caspase-8 and FADD-like IL-1β-converting enzyme (FLICE)-like inhibitory proteins (FLIPs)), which induces caspase-8 homodimerization and activation, which activates the executioner caspases (caspase-3, caspase-6, and caspase-7), with resulting apoptosis [21,22]. Activation of the caspase cascade results in the cleavage of a number of important cellular proteins, known as the “cell-death substrates” such as actin, nuclear lamins, inhibitor of the caspase-activated DNase (ICAD), and RAS homologue (RHO)-associated coiled-coil-containing protein kinase 1 (ROCK1). The dying cells express “eat-me” signals, such as phosphatidyl serine and different surface sugars, which allow the dying cells to be removed by phagocytes [23].

The intrinsic apoptotic pathway is triggered by TCR stimulation, DNA damage, endoplasmic reticulum (ER) stress, hormones, or cytokine deprivation. The extrinsic and intrinsic apoptotic pathways converge at the level of the effector caspases, as activated caspase-8 is also able to cause cleavage of the B-cell lymphoma 2(BCL-2)-family protein BH3-interacting-domain death agonist (BID) to generate truncated BID (tBID). tBID induces the pro-apoptotic functions of the mitochondria by causing the oligomerization of BAX (BCL-2-associated X protein) and/or BAK (BCL-2 antagonist/killer). Pro-apoptotic proteins of the BCL-2 family can be classified according to the number of BCL-22 homology (BH1-4) domains in their sequence. BAX and BAK, for example, are multi-domain proteins containing the BH1, BH2 and BH3 domains. The oligomerization of effectors (BAX–BAX, BAX–BAK) on the mitochondrial outer membrane (MOM) provokes its permeabilization [24], with resulting: (a) mitochondrial dysfunction including reactive oxygen species (ROS) formation; (b) release of cytochrome C and formation of the apoptosome [17], followed by the activation of pro-caspase-9, which is thus able to cleave the downstream effectors pro-caspase-3, pro-caspase-6 and pro-caspase-7; and (c) release of other pro-apoptotic molecules (endonuclease G, second mitochondria-derived activator of caspases (SMAC), all of which ensure the cell’s demise) (Figure 1).

Life or death pathways in T-cells are dictated by the balance of anti-apoptotic factors and pro-apoptotic factors. Examples of anti-apoptotic factors blocking downstream caspases activation include the caspase-8 (FLICE)-like inhibitory protein (FLIP) for the extrinsic pathway [25]. FLIP, a protein structurally related to caspase-8 but without protease activity, forms a heterodimer that prevents caspase-8– mediated apoptosis. The caspase-8–FLIP heterodimer predominates in cells with higher FLIP expression, which is induced on NF-κB activation upon apoptotic signaling. Scientific evidence suggests that high concentrations of cellular FLIP (cFLIP) long isoform (cFLIPL) inhibit the activation of pro-caspase-8 at the CD95 DISC by blocking its processing [26], whereas low concentrations of cFLIPL facilitate the cleavage of pro-caspase-8 [27,28].

The intrinsic apoptotic pathway is also regulated by the balance of pro-apoptotic and anti-apoptotic members of the BCL-2 family, by other mitochondria derived molecules, and by other caspase inhibitors. BCL-2 inhibitor of apoptosis inhibits the apoptotic process in several steps, by controlling calcium flux [29] and binding and inhibiting pro-apoptotic proteins (e.g., BAX/BAK) [30,31]. Other studies also suggest a role in the regulation of the activity of beclin-1 to initiate autophagy [32], and in the regulation of cytoplasmic levels of acetyl coenzyme A (acetyl-CoA) as a substrate for protein α-acetylation as a signaling molecule associated with apoptotic sensitivity [33]. X-linked inhibitor of apoptosis protein (XIAP), a member of the inhibitor of apoptosis family of proteins (IAPs), binds to and inhibits caspase-3, -7 and -9 [34]. Interestingly, molecules released by the mitochondria, such as SMAC, are able to inhibit IAP mediated inhibition of caspases. Additionally, certain BH3-only proteins termed “activators”, notably BID and BCL-2-interacting mediator of cell death (BIM or BCL2L11), can bind transiently to BAX and induce its activation, whereas the others, termed ‘‘sensitizers’’ (e.g., BCL-2-associated death promoter (BAD)), act instead by freeing the activators or BAX from pro-survival relatives (e.g., anti-apoptotic molecules such as BCL-2).

The same molecule can act as either pro-apoptotic or anti-apoptotic depending on the type of activation signal involved. For example, the activation of caspases-8, -3 and/or -7 through TCR stimulation rather than cell death pathways can exert a pro-survival function [35,36]. Earlier evidence suggests the possibility of a differential activation of the intrinsic versus the extrinsic apoptotic pathway. In this research, the authors determined that the presence of high levels of CD95 resulted in a high amount of caspase-8 activation, with resulting activation of caspase-3, whereas low levels of CD95 resulted in a lower amount of caspase-8 activation with predominance of the mitochondrial pathway over caspase-3 activation [37]. CD95 ligand (FASL) expression is restricted to cytotoxic T-lymphocytes, T helper 1 cells and natural killer cells, whereas CD95 (FAS) is widely expressed by most cell types in various tissues, and under physiologic conditions FASL-mediated apoptosis also contributes to the maintenance of tissue homeostasis. Additionally, dysregulation of this balance has been proposed to have a role in the development of GVHD in a mouse model of allo-HSCT [38].

The regulation of life and death of T-cells uses molecular mechanisms that may be different in distinct T-cell populations, depending on their state of activation. Studies in cell lines and primary human and murine T-cells show that while full length hematopoietic progenitor kinase 1 (HPK1) leads to activation of the NF-κB complex upon TCR stimulation, whereas its proteolytic fragment HPK1-C blocks NF-κB activation after TCR ligation [39]. The authors proposed this as a novel mechanism of sensitization of T-lymphocytes towards AICD by suppression of NF-κB, with HPK1 as a novel life/death switch in T-lymphocytes.

Apoptosis is a clean process without inflammation or tissue destruction because apoptotic cells are rapidly engulfed by phagocytes (efferocytosis), due to the translocation of phosphatidylserine from the inner leaflet to the outer leaflet of the plasma membrane as a consequence of caspases activation. After being engulfed by the phagocytes, apoptotic cells are transported to lysosomes, where their components are degraded into building units for re-use [40]. Internalization of apoptotic cells exerts immune-regulatory effects on target antigen presenting cells (APCs), thus preventing immune responses against self-tissue [41].

## 3. Exploiting Apoptotic Pathways to Prevent or Treat Graft-versus-Host-Disease

Several laboratories have tested the possibility of promoting antigen specific tolerance for the therapy of GVHD (and also graft rejection, or autoimmune disorders), by developing methodologies that mimic the mechanisms by which the immune system maintains peripheral tolerance in the steady state. The principle is that administration of apoptotic donor cells would target host APCs and down-regulate its immunogenic function to control GVHD [42]. Interestingly, procedures such as extracorporeal photopheresis (ECP) are associated with induction of host/recipient immunosuppressive dendritic cells (DCs) by the clearance of the ECP-treated apoptotic cells and with increased number or function of CD4 T-regulatory cells [43,44]. Based on these observations, some authors have proposed the hypothesis that infusion of host reactive T-cells undergoing immunogenic cell death provides a source of host-reactive antigens (TCR-derived peptides) plus signals that activate host APCs, resulting in the generation of anti-clonotypic CD8 T-cell responses that eliminate the pathogenic T-cells responsible for GVHD [45].

Some investigated strategies aimed at blocking the interaction between molecules associated with T-cell function, potentially resulting in reduced GVHD without compromised graft-versus-tumor (GVT) activity. One such strategy included blocking fibroblast growth factor-inducible 14 (FN14) interaction with tumor necrosis factor (TNF)-like weak inducer of apoptosis (TWEAK). This investigation was performed in Balb/c mice (H-2d) engrafted with allogeneic bone marrow and T-cells from black 6 (B6) donor mice (H-2b), as well as murine lymphoma cells. Tweak-Fn14 inhibition was accomplished using an anti-FN14-IgG1 antibody variant with compromised antibody-dependent cellular cytotoxicity (ADCC) activity. This inhibition reduced the incidence and severity of gastrointestinal GVHD. Importantly, Fn14 blockade showed no negative effect on GVT activity [46]. To date, only anti-TWEAK monoclonal antibodies have been investigated in clinical trials [47,48].

Another strategy involved the infusion of TRAIL overexpressing donor T-cells in mice with resulting increased induced apoptosis of alloreactive T-cells with reduced GVHD in a death receptor 5 (DR5) dependent manner, with an unexpected increase of in vitro and in vivo GVT effect. TRAIL^+^ T-cells led to 100% survival of mice with lymphoma and minimal GVHD, while mice receiving control T-cells all succumbed to lymphoma and GVHD. This positive effect was reproduced with human T-cells with TRAIL overexpression leading to enhanced cytolysis of tumor cells and alloreactive T-cells, as compared with control T-cells. This combination of decreased GVHD and increased GVT effect makes this genetic engineering of donor T-cells in the allo-HSCT setting a potentially promising cell therapy [49].

Ni et al. reported on the temporary depletion of donor CD4^+^ T-cells immediately after allo-HSCT using a single dose of human anti-CD4 monoclonal antibody IT1208, currently under clinical investigation for treatment of advanced solid tumors and in vivo murine leukemia and lymphoma models. This approach led to increased interferon gamma production and Programmed death-ligand 1 (PD-L1) upregulation of PD-L1 on recipient GVHD-targeted tissues. The interaction with PD-1 expressing CD8^+^ T-cells and PD-L1^+^ target tissues of GVHD resulted in T-cell exhaustion and apoptosis, thereby preventing acute GVHD but not chronic GVHD, which was associated with reconstitution of donor CD4^+^ T-cells beginning by day 21 post HSCT. However, three doses of anti-CD4 monoclonal antibody on days 0, 14, and 28 prevented both acute and chronic GVHD, and subsequently recovered donor CD4^+^ T-cells did not cause chronic GVHD. Conversely, PD-L1 upregulation on donor CD8^+^ T-cell and subsequent interaction with CD80 on host lymphoid tissues led to increased CD8^+^ T-cell survival and GVT effect [50].

Leclerc et al. have demonstrated that blocking TNF/Tumor Necrosis Factor Receptor 2(TNF-R2) interaction on T-regulatory cells (T-regs) prevented GVHD without inhibiting the GVT effect. This was accomplished infusing a TNF-R2 mAb to C57BL/6 mice allografted with bone, marrow, T-cells, with or without HY-antigen specific T-regs from sex mismatched cells from sex mismatched C57 black 6 (C57BL/6) mice (semi-allogeneic model). Targeting TNF/TNF-R2 interaction represents an opportunity to efficiently modulate alloreactivity in allo-HSCT to either exacerbate it for a powerful antileukemic effect or reduce it to control GVHD [51].

Mizraih et al. found that ex vivo exposure of mobilized peripheral blood hematopoietic progenitor cells to FAS-ligand (FASL) and TNF-α apoptotic ligands could inhibit GVHD without impairing GVT. This was investigated in non-obese diabetic mice with severe combined immunodeficiency disease (NOD.SCID) engrafted with human mobilized peripheral blood hematopoietic progenitor cells and the human colon carcinoma HT29 cell line, where functional assays revealed that the death receptors’ modulated graft composition as compared with incubation in medium, without detectable quantitative variations. Apparently, pre-treating mobilized peripheral blood hematopoietic progenitor cells with toxic doses of FASL and TNF-α did not affect their proliferative and engraftment potential [52]. All in one, these strategies open the door to potential clinical investigation of strategies aiming at increasing the GVT effect while containing GVHD.

The deeper understanding of the molecular pathways of apoptosis in T-cells may open the door to innovative therapeutic strategies to prevent or eliminate GVHD, autoimmunity, and eventually overcome the inhibitory effects of the tumor on T-cell mediated immune responses.

## 4. Apoptosis by Ligand-Mediated Dimerization for Graft-versus-Host-Disease

Spencer and colleagues, using cell permeable synthetic ligands that can bind FK506 Binding Protein 12 (FKBP12) [53], demonstrated the ability to control cellular signaling pathways through ligand-mediated dimerization of intracellular proteins [54].

In order eliminate the immunosuppressive and toxic effect of calcineurin inhibition [55], FK506 variants that have impaired calcineurin binding activity were generated [54]. Dimeric forms of variant FK506 can induce intracellular dimerization and signaling of engineered chimeric proteins that contain FKBP12 domains whilst monomeric forms block this signaling [54]. A further improvement to the system was introduced a few years later by Clackson and colleagues, who redesigned the interface between FKBP12 and the synthetic ligand to reduce the potential for ligand interaction with endogenous FKBP12, which may interfere with the physiological function of FKBP12 and potentially lead to compound sequestration and blunted potency [10]. This is achieved by creating a specificity binding pocket in FKBP12 by substituting phenylalanine with the smaller valine residue (FKBP12-F36V) and introducing an ethyl “bump” into the FKBP12 ligand, resulting in subnanomolar affinity but 1000-fold selectivity for FKBP12-F36V [10].

The first reports of dimerization-induced apoptosis involved inducible Fas receptors (iFas) [56,57]. Other systems soon followed. These included the use of the death effector domain of FADD [58,59], iC1 [58,60,61], iC3 [58,62], iC8 [63], ΔiC8 [58,61,64,65,66,67], iC9 [58,68,69], ΔiC9 [61] and inducible BAX (iBAX) [70] with variable efficacy in inducing cell death ranging from 30% to ~90%.

In designing a gene therapy strategy, it is important to consider the effect of the over-expression of the pro-apoptotic candidate gene on cell survival, as, for example, mere overexpression of the wild type BAX molecule has been demonstrated to significantly limit cell viability [71]. Moreover, the impact of the removal of the endogenous activatory domain from pro-apoptotic genes needs to be considered. For example, enforced dimerization of caspase-8 without endogenous prodomain (ΔiC8) [58,61,64,65,66,67] led to increased cell death as compared to the full-length molecule [61,63], without apparent baseline toxicity, likely due to by-passing the first activation and cleavage step.

Different requirements on cleavage and dimerization for each molecule, together with the different design of the transgene constructs, may explain why enforced dimerization of some molecules, such as caspase-3, resulted in inducible cell death in some experiences [52,53], but not in others [61]. Additionally, co-expression of an initiator caspase (e.g., caspase-9) with an executioner caspase, such as caspase-3, could result in a synergistic/additive effect by providing a higher amount of downstream activatable substrate [62]. Deleting endogenous pro-domain(s) could be more problematic in molecules for which their truncated form would be expected to lead to spontaneous dimerization and activation of cell death pathways, as has been demonstrated for some isoforms of truncated BAX [72]. The induction of apoptosis by membrane proximal molecules may potentially be more susceptible to downstream inhibitors of apoptosis such as FLIP, BCL-2 and B-cell leukemia XL (BCL-XL). The use of terminal effector molecules, such as caspase-3 or caspase-7, would be ideal, but, in previous limited experiences, it was difficult to express these molecules in primary human T-cells at functional levels [69]. This led to the development of ΔiC9 as a candidate for inducible apoptosis of human T-cells for therapeutic means.

### Inducible Caspase9 (ΔiC9) Suicide Gene

Caspase-9 is part of the intrinsic apoptotic pathway. It is activated by the release of cytochrome C from damaged mitochondria through its interaction with apoptotic protease-activating factor 1 (APAF-1). Caspase-9 can then activate caspase-3 and the other terminal effector molecules. The optimized ΔiC9 molecule consists of one FKBP12-F36V binding domain linked, via a Ser-Gly-Gly-Gly-Ser linker, to a caspase-9 molecule. The caspase recruitment domain (CARD) is removed from ΔiC9 because its physiological function of effecting caspase-9 dimerization is now superfluous [69].

Infusion of *ΔiC9* gene modified donor T-cells has resulted in significant in vivo expansion and persistence, both in patients who developed GVHD and in patients who did not. Administration of a single dose of a non-therapeutic dimerizing agent resulted in ≥90% gene modified cell elimination within hours, with resolution of acute GVHD within 24 h [6]. Interestingly, residual T-cells were able to re-expand, persist long term, and exert anti-viral and anti-fungal activity, whilst not resulting in any additional acute GVHD [7,8]. The incomplete elimination of ΔiC9 transduced cells could be explained by several mechanisms, such as the selective elimination of cells with higher transgene expression [6], epigenetic modulation of promoters proximal to vector insertion sites [73], or selection of cells with qualitatively or quantitatively higher anti-apoptotic factor expression [74]. The emergence of mutation in the transgene has not been observed, however [73]. Despite its incomplete elimination, the infusion of *ΔiC9* suicide gene modified T-cells after allo-HSCT has resulted in effective control of GVHD, and, although the number of patients treated thus far is low, it has the potential of reducing GVHD-related morbidity and mortality.

Considering the sigmoid dose-response curve of the homodimerizer on ΔiC9, one current research interest involves dose reduction of the dimerizer to activate the ΔiC9 switch, in order to control GVHD without abrogating it, thereby potentially preserving anti-tumor immunity to the highest degree.

The ΔiC9 strategy is now being investigated in different settings, such as in patients with relapsed hematologic malignancies after related transplants (NCT01744223, 5 December 2012), as well as add-back after haploidentical transplants in patients with non-malignant conditions (NCT02065869 13 February 2014).

Contrary to the *ΔiC9* suicide gene, the HSV-TK, being almost completely human derived, proved immunogenic, especially in patients with a higher degree of T-cell immune reconstitution, with limited persistence of HSV-TK cells [75]. Additionally, GCV-resistant truncated HSV-TK forms have been observed [76]. In both the HSV-TK and ΔiC9 studies, infusion of suicide gene modified cells aided non-gene modified T-cell immune reconstitution [6,77,78], likely secondary to interleukin-7 secretion by the gene modified cells [79]. The lack of further acute GVHD manifestations in these studies may suggest either (i) complete elimination of allo-reactive cells; or (ii) development of peripheral tolerance. Additionally, the incidence of chronic GVHD was low in the HSV-TK T-cell studies, and absent in the ΔiC9 trial [6,7].

## 5. Caspase-Independent Cell Death in T-Cells: Necroptosis

Necroptosis is a form of caspase-independent cell death in T-cells occurring especially during viral or bacterial infections, with the purpose to kill pathogen-infected cells before the invading pathogen proliferates. Necroptosis is a form of molecular programmed necrosis characterized by swelling of the organelles and plasma membrane rupture (as opposed to cell shrinkage, chromatin condensation, and cellular fragmentation as seen in apoptosis), triggered by a variety of stimuli, including infectious agents, intracellular disturbances (e.g., ATP depletion), cell surface death receptors’ activation, such as toll-like receptors 3 and 4, or TNFR1, and other factors as reviewed in detail elsewhere [80]. Unlike apoptosis, in which several of the highly immunogenic intracellular proteins are sequestered in the dead cell, necroptosis is accompanied by the release of cellular contents from the dying cells, which act as damage-associated molecular patterns (DAMPs) to stimulate pro-inflammatory processes and recruitment of immune cells.

TNF produced by macrophages has the ability to induce apoptosis or necrosis, especially in the presence of inhibitors of protein and DNA or RNA synthesis. TNF kills cells by necroptosis when the apoptosis pathway is inhibited; in fact, activated caspase-8 exerts a blocking effect on necroptosis. When cells are infected by a virus or bacteria, their transcriptional or translational machinery is inhibited; in addition, viruses and bacteria often encode molecules that inhibit apoptosis, thereby sensitizing the target cells to TNF-induced necroptosis.

**Figure 2:**
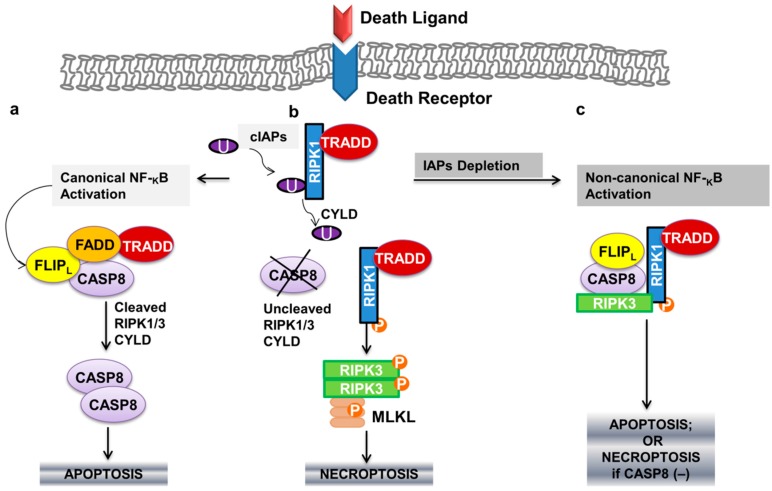
(**a**) Apoptotic pathway of programmed cell death in T-lymphocytes and its inter-relation with (**b**,**c**) the programmed necrotic cell death (necroptotic pathway). Arrows indicate activation. cIAPs: cellular inhibitor of apoptosis family of proteins; U: ubiquitination; RIPK1: receptor-interacting protein kinase 1; TRADD: TNFR1-associated death domain; FLIP_L_: FLICE-like inhibitory proteins (long isoform); FADD: FAS-associated death domain; CASP: caspase; RIPK3: receptor-interacting protein kinase 3; CYLD: cylindromatosis; MLKL: mixed lineage kinase domain-like protein; P: phosphorylation.

Recruitment of TRADD by stimulation of TNFR1, as described in the previous paragraphs, also attracts receptor-interacting protein kinase 1 (RIPK1), forming TRADD-dependent receptor complex I. RIPK1 is then subject to Lys63 polyubiquitylation by IAP1 or cIAP2, activating NF-κB, with resulting upregulation of anti-apoptotic genes such as FLIPL [81,82]. A more detailed description of additional adaptor molecules involved can be found in a nice recently published article [80]. In cells undergoing apoptosis, cylindromatosis (CYLD) would remove Lys63-linked polyubiquitins from RIPK1 [83], rendering complex I unstable and allowing RIPK1 to dissociate from the plasma membrane and interact with TRADD-dependent complex IIa; in this complex FLIPL and pro-caspase 8 form a heterodimeric caspase that cleaves and inactivates RIPK1 (and RIPK3), as well as CYLD, to prevent necroptosis [84] (Figure 2a). However, when caspase-8 is inhibited, the RIP homotypic interaction motif (RHIM) domains of RIPK1 and RIPK3 associate in microfilament-like complexes called necrosomes, an amyloid-like structure that acts as the transducer of the necroptotic signal [85]. RIPK3 is thus able to phosphorylate a protein called mixed lineage kinase domain-like protein (MLKL), which then translocates to the plasma membrane, causing damage to execute necrosis [86]. (Figure 2b). This results in a late wave of JUN N-terminal kinase (JNK) activation, ROS production and the induction of necroptosis [86]. Additionally, oligomerized MLKL translocates to the plasma membrane mediating TNF-induced necroptosis in a calcium influx-dependent way [87]. Importantly, IAP degradation (for example, in the presence of SMAC), will result in non-canonical NF-κB activation, with formation of a TRADD independent complex between RIPK1/3, FADD and FLIPL (ripoptosome or complex IIb). RIPK1-dependent complex IIb can induce both necroptosis and apoptosis, which is dependent on the absence or presence of caspase-8 activity, respectively (Figure 2c).

Necroptosis is involved in lymphocyte homeostasis [88], as well as in the organ damage seen in several inflammatory conditions, including ischemic organ injury [80,88]; therefore, therapeutic intervention to block this process are under investigation. Programmed necrotic cell death is also the mediator of cell death ensuing after the administration of some chemotherapeutic drugs [89]. Although further investigations are needed to define the role of necroptosis in cancer, inducing RIPK-3 dependent necrosis is an attractive strategy to circumvent apoptosis resistance from chemotherapeutic or targeted agents on cancer cells [88,90].

### Exploiting Necroptosis for Inducible Cell Death

Several groups have designed inducible dimerization systems in order to more fully understand the molecular mechanisms leading to activation of necroptosis [59]. This system could also eventually be exploited for potential therapeutic means. Orozco et al. [91] cloned a chimeric protein of murine RIPK3 fused to one or two copies of FKBP12-F36V. The authors found that adding 1-2 FKBP-F36V domains before full length RIPK3 induced significant cell death (~70–80% cell death after 6 h of incubation with 50 nM of homodimerizer agent), whereas only when two FKBP12–F36V domains were added to RIPK3 with deleted RHIM domain, cell death was induced to an analogous extent.

Contrarily, Wu et al. [92] demonstrated efficient inducible cell death even when using a single drug binding domain in the absence of the RHIM domain, if the drug binding domain was appended to the N-terminus, rather than to the C-terminus of RIPK3. In the experience of Wu et al., a heterodimerization system was used, based on an FKBP and an FRB-T2098L drug binding domain, activated by a heterodimerizer agent, instead.

Enforced dimerization of MLKL through addition of a gyrase domain also resulted in potent inducible cell death in vitro [93]. Being a molecule more downstream in the necroptotic pathway, MLKL may be susceptible to a lesser number of inhibitory factors.

Inducible necroptosis could be exploited for the safety of T-cells or other cellular therapeutics, albeit dedicated validation experiments would need to be accomplished for this goal.

Models of inducible necroptosis and inducible apoptosis also offer a framework for studying the increasingly recognized interactions between the two processes. A better understanding of this relationship could open the door to future research aiming at exploiting these important cell death pathways for cellular therapy, as well as anti-cancer treatments. Although enforced dimerization of inducible proteins of the apoptotic pathway or the necroptotic pathway led to impressive inducible cell elimination, in order to grant complete elimination of the gene modified cells, one potential strategy involves the combination of two inducible molecules. Perhaps, combining one inducible molecule from the apoptotic pathway and one inducible molecule from the necroptotic pathway could counteract immune-evasion strategies characteristic of each death process.

## 6. Conclusions

Suicide gene strategies have the potential to increase the safety and the clinical applicability of novel cellular therapies. The infusion of suicide gene modified T-cells after allo-HSCT has resulted in effective control of GVHD, which can substantially reduce GVHD-related non-relapse morbidity and mortality. Current research strategies aim to maximize the positive impact on both non-relapse and relapse mortality in this setting.

Suicide gene application can also be beneficial for the safety of other T-cell applications, such as the infusion of chimeric antigen receptor (CAR) redirected T-cells [94,95,96], and also other cell types, such as inducible pluripotent stem cells (iPSC), both of which are emerging rapidly as extremely promising treatments for cancer or regenerative medicine [97,98,99]. They do, however, carry the intrinsic risks of excess proliferation [100,101], insertional mutagenesis [100,101,102,103,104,105,106], and in the case of CAR-T specifically, off-target side effects resulting in severe cytokine release syndrome (CRS) and potentially fatal organ damage and death [107]. Currently, it is not possible to predict the type or degree of toxicities that may occur. For example, injection of even unmodified autologous hematopoietic stem cells (HSC) into the kidneys of a patient with renal failure was associated with the development of angiomyeloproliferative lesions that required nephrectomy [108]. Autologous stem cells derived from adipose tissue and injected intravitreally for macular degeneration were associated with worsening vision in three people, two of whom became legally blind [109]. Gene-modified HSC infused into patients with monogenic disease [102,103,105,106,110] resulted in leukemia from insertional mutagenesis in up to 70% of patients, and even a higher risk may arise with the use of iPSC. For example, a patient developed glioneuronal multifocal brain cancer after the infusion of donor fetal-derived neuronal stem cells [100]. These detrimental effects could be potentially alleviated by employing a cellular suicide gene strategy in which a gene is inserted into the therapeutic cell and can then be activated “on demand” to cause cell death.

Considering that there are many human diseases associated with abnormal cell death processes, especially autoimmune, inflammatory conditions, and cancer, detailed findings about cell death pathways will also contribute to the understanding of disease pathogenesis, with the potential of developing novel forms of treatment. As a matter of fact, many agents are under active investigation targeting anti-apoptotic factors and cell survival proteins for the treatment of patients with cancer, with the goal of inhibiting cancer cell survival and potentially reversing chemoresistance [111].

In summary, investigations regarding the balance between cell survival and cell death represent a fascinating chapter of investigative medicine, with multiple potential clinical applications.

## References

[1] Singh A.K., McGuirk J.P. (2016). Allogeneic stem cell transplantation: A historical and scientific overview. Cancer Res..

[2] Li Pira G., di Cecca S., Montanari M., Moretta L., Manca F. (2016). Specific removal of alloreactive T-cells to prevent GVHD in hemopoietic stem cell transplantation: Rationale, strategies and perspectives. Blood Rev..

[3] Kanakry C.G., Fuchs E.J., Luznik L. (2016). Modern approaches to HLA-haploidentical blood or marrow transplantation. Nat. Rev. Clin. Oncol..

[4] Jones B.S., Lamb L.S., Goldman F., di Stasi A. (2014). Improving the safety of cell therapy products by suicide gene transfer. Front. Pharmacol..

[5] Weissinger E.M., Borchers S., Silvani A., Provasi E., Radrizzani M., Beckmann I.K., Benati C., Schmidtke J., Kuehnau W., Schweier P. (2015). Long term follow up of patients after allogeneic stem cell transplantation and transfusion of HSV-TK transduced T-cells. Front. Pharmacol..

[6] Di Stasi A., Tey S.K., Dotti G., Fujita Y., Kennedy-Nasser A., Martinez C., Straathof K., Liu E., Durett A.G., Grilley B. (2011). Inducible apoptosis as a safety switch for adoptive cell therapy. N. Engl. J. Med..

[7] Zhou X., di Stasi A., Tey S.K., Krance R.A., Martinez C., Leung K.S., Durett A.G., Wu M.F., Liu H., Leen A.M. (2014). Long-term outcome after haploidentical stem cell transplant and infusion of T-cells expressing the inducible caspase 9 safety transgene. Blood.

[8] Zhou X., Dotti G., Krance R.A., Martinez C.A., Naik S., Kamble R.T., Durett A.G., Dakhova O., Savoldo B., di Stasi A. (2015). Inducible caspase-9 suicide gene controls adverse effects from alloreplete T-cells after haploidentical stem cell transplantation. Blood.

[9] Moolten F.L. (1986). Tumor chemosensitivity conferred by inserted herpes thymidine kinase genes: Paradigm for a prospective cancer control strategy. Cancer Res..

[10] Clackson T., Yang W., Rozamus L.W., Hatada M., Amara J.F., Rollins C.T., Stevenson L.F., Magari S.R., Wood S.A., Courage N.L. (1998). Redesigning an FKBP-ligand interface to generate chemical dimerizers with novel specificity. Proc. Natl. Acad. Sci. USA.

[11] Hildeman D.A., Zhu Y., Mitchell T.C., Bouillet P., Strasser A., Kappler J., Marrack P. (2002). Activated T-cell death in vivo mediated by proapoptotic BCL-2 family member bim. Immunity.

[12] Mercep M., Weissman A.M., Frank S.J., Klausner R.D., Ashwell J.D. (1989). Activation-driven programmed cell death and T-cell receptor zeta eta expression. Science.

[13] Kerr J.F., Wyllie A.H., Currie A.R. (1972). Apoptosis: A basic biological phenomenon with wide-ranging implications in tissue kinetics. Br. J. Cancer.

[14] Krammer P.H., Arnold R., Lavrik I.N. (2007). Life and death in peripheral T-cells. Nat. Rev. Immunol..

[15] Sprent J., Tough D.F. (2001). T-cell death and memory. Science.

[16] Lavrik I., Golks A., Krammer P.H. (2005). Death receptor signaling. J. Cell Sci..

[17] Lavrik I.N., Golks A., Krammer P.H. (2005). Caspases: Pharmacological manipulation of cell death. J. Clin. Investig..

[18] Thornberry N.A., Lazebnik Y. (1998). Caspases: Enemies within. Science.

[19] Krammer P.H. (2000). CD95’s deadly mission in the immune system. Nature.

[20] Ashkenazi A., Dixit V.M. (1998). Death receptors: Signaling and modulation. Science.

[21] Boatright K.M., Renatus M., Scott F.L., Sperandio S., Shin H., Pedersen I.M., Ricci J.E., Edris W.A., Sutherlin D.P., Green D.R. (2003). A unified model for apical caspase activation. Mol. Cell.

[22] Muzio M., Stockwell B.R., Stennicke H.R., Salvesen G.S., Dixit V.M. (1998). An induced proximity model for caspase-8 activation. J. Biol. Chem..

[23] Igney F.H., Krammer P.H. (2002). Immune escape of tumors: Apoptosis resistance and tumor counterattack. J. Leukoc. Biol..

[24] Kroemer G., Galluzzi L., Brenner C. (2007). Mitochondrial membrane permeabilization in cell death. Physiol. Rev..

[25] Bentele M., Lavrik I., Ulrich M., Stosser S., Heermann D.W., Kalthoff H., Krammer P.H., Eils R. (2004). Mathematical modeling reveals threshold mechanism in CD95-induced apoptosis. J. Cell Biol..

[26] Krueger A., Schmitz I., Baumann S., Krammer P.H., Kirchhoff S. (2001). Cellular flice-inhibitory protein splice variants inhibit different steps of caspase-8 activation at the CD95 death-inducing signaling complex. J. Biol. Chem..

[27] Micheau O., Thome M., Schneider P., Holler N., Tschopp J., Nicholson D.W., Briand C., Grutter M.G. (2002). The long form of FLIP is an activator of caspase-8 at the FAS death-inducing signaling complex. J. Biol. Chem..

[28] Chang D.W., Xing Z., Pan Y., Algeciras-Schimnich A., Barnhart B.C., Yaish-Ohad S., Peter M.E., Yang X. (2002). C-FlIP(L) is a dual function regulator for caspase-8 activation and CD95-mediated apoptosis. EMBO J..

[29] Zeng S., Liu W., Nie F.F., Zhao Q., Rong J.J., Wang J., Tao L., Qi Q., Lu N., Li Z.Y. (2009). Lyg-202, a new flavonoid with a piperazine substitution, shows antitumor effects in vivo and in vitro. Biochem. Biophys. Res. Commun..

[30] Minn A.J., Kettlun C.S., Liang H., Kelekar A., Vander Heiden M.G., Chang B.S., Fesik S.W., Fill M., Thompson C.B. (1999). BCL-XL regulates apoptosis by heterodimerization-dependent and -independent mechanisms. EMBO J..

[31] Mathai J.P., Germain M., Marcellus R.C., Shore G.C. (2002). Induction and endoplasmic reticulum location of BIK/NBK in response to apoptotic signaling by E1A and p53. Oncogene.

[32] Rosenfeldt M.T., Nixon C., Liu E., Mah L.Y., Ryan K.M. (2012). Analysis of macroautophagy by immunohistochemistry. Autophagy.

[33] Andersen J.L., Kornbluth S. (2011). Meeting the (N-terminal) end with acetylation. Cell.

[34] Deveraux Q.L., Takahashi R., Salvesen G.S., Reed J.C. (1997). X-linked IAP is a direct inhibitor of cell-death proteases. Nature.

[35] Kennedy N.J., Kataoka T., Tschopp J., Budd R.C. (1999). Caspase activation is required for T-cell proliferation. J. Exp. Med..

[36] Su H., Bidere N., Zheng L., Cubre A., Sakai K., Dale J., Salmena L., Hakem R., Straus S., Lenardo M. (2005). Requirement for caspase-8 in NF-κB activation by antigen receptor. Science.

[37] Scaffidi C., Fulda S., Srinivasan A., Friesen C., Li F., Tomaselli K.J., Debatin K.M., Krammer P.H., Peter M.E. (1998). Two CD95 (APO-1/FAS) signaling pathways. EMBO J..

[38] Tsukada N., Kobata T., Aizawa Y., Yagita H., Okumura K. (1999). Graft-versus-leukemia effect and graft-versus-host disease can be differentiated by cytotoxic mechanisms in a murine model of allogeneic bone marrow transplantation. Blood.

[39] Brenner D., Golks A., Kiefer F., Krammer P.H., Arnold R. (2005). Activation or suppression of NF-κB by HPK1 determines sensitivity to activation-induced cell death. EMBO J..

[40] Nagata S., Tanaka M. (2017). Programmed cell death and the immune system. Nat. Rev. Immunol..

[41] Morelli A.E., Larregina A.T. (2016). Concise review: Mechanisms behind apoptotic cell-based therapies against transplant rejection and graft versus host disease. Stem Cells.

[42] Saas P., Gaugler B., Perruche S. (2010). Intravenous apoptotic cell infusion as a cell-based therapy toward improving hematopoietic cell transplantation outcome. Ann. N. Y. Acad. Sci..

[43] Lamioni A., Parisi F., Isacchi G., Giorda E., di Cesare S., Landolfo A., Cenci F., Bottazzo G.F., Carsetti R. (2005). The immunological effects of extracorporeal photopheresis unraveled: Induction of tolerogenic dendritic cells in vitro and regulatory T-cells in vivo. Transplantation.

[44] Maeda A., Schwarz A., Bullinger A., Morita A., Peritt D., Schwarz T. (2008). Experimental extracorporeal photopheresis inhibits the sensitization and effector phases of contact hypersensitivity via two mechanisms: Generation of IL-10 and induction of regulatory T-cells. J. Immunol..

[45] French L.E., Alcindor T., Shapiro M., McGinnis K.S., Margolis D.J., Porter D., Leonard D.G., Rook A.H., Foss F. (2002). Identification of amplified clonal T-cell populations in the blood of patients with chronic graft-versus-host disease: Positive correlation with response to photopheresis. Bone Marrow Transplant..

[46] Chopra M., Brandl A., Siegmund D., Mottok A., Schafer V., Biehl M., Kraus S., Bauerlein C.A., Ritz M., Mattenheimer K. (2015). Blocking TWEAK-FN14 interaction inhibits hematopoietic stem cell transplantation-induced intestinal cell death and reduces GVHD. Blood.

[47] Wisniacki N., Amaravadi L., Galluppi G.R., Zheng T.S., Zhang R., Kong J., Burkly L.C. (2013). Safety, tolerability, pharmacokinetics, and pharmacodynamics of anti-TWEAK monoclonal antibody in patients with rheumatoid arthritis. Clin. Ther..

[48] Lassen U.N., Meulendijks D., Siu L.L., Karanikas V., Mau-Sorensen M., Schellens J.H., Jonker D.J., Hansen A.R., Simcox M.E., Schostack K.J. (2015). A phase i monotherapy study of RG7212, a first-in-class monoclonal antibody targeting tweak signaling in patients with advanced cancers. Clin. Cancer Res. Off. J. Am. Assoc. Cancer Res..

[49] Ghosh A., Dogan Y., Moroz M., Holland A.M., Yim N.L., Rao U.K., Young L.F., Tannenbaum D., Masih D., Velardi E. (2013). Adoptively transferred trail+ T-cells suppress GVHD and augment antitumor activity. J. Clin. Investig..

[50] Ni X., Song Q., Cassady K., Deng R., Jin H., Zhang M., Dong H., Forman S., Martin P.J., Chen Y.Z. (2017). PD-L1 interacts with CD80 to regulate graft-versus-leukemia activity of donor CD8+ T-cells. J. Clin. Investig..

[51] Leclerc M., Naserian S., Pilon C., Thiolat A., Martin G.H., Pouchy C., Dominique C., Belkacemi Y., Charlotte F., Maury S. (2016). Control of gvhd by regulatory T-cells depends on TNF produced by T-cells and TNFR2 expressed by regulatory T-cells. Blood.

[52] Mizrahi K., Yaniv I., Ash S., Stein J., Askenasy N. (2014). Apoptotic signaling through FAS and TNF receptors ameliorates GVHD in mobilized peripheral blood grafts. Bone Marrow Transplant..

[53] Schreiber S.L. (1991). Chemistry and biology of the immunophilins and their immunosuppressive ligands. Science.

[54] Spencer D.M., Wandless T.J., Schreiber S.L., Crabtree G.R. (1993). Controlling signal transduction with synthetic ligands. Science.

[55] Clipstone N.A., Crabtree G.R. (1992). Identification of calcineurin as a key signalling enzyme in T-lymphocyte activation. Nature.

[56] Spencer D.M., Belshaw P.J., Chen L., Ho S.N., Randazzo F., Crabtree G.R., Schreiber S.L. (1996). Functional analysis of FAS signaling in vivo using synthetic inducers of dimerization. Curr. Biol..

[57] Belshaw P.J., Spencer D.M., Crabtree G.R., Schreiber S.L. (1996). Controlling programmed cell death with a cyclophilin-cyclosporin-based chemical inducer of dimerization. Chem. Biol..

[58] Fan L., Freeman K.W., Khan T., Pham E., Spencer D.M. (1999). Improved artificial death switches based on caspases and FADD. Hum. Gene Ther..

[59] Grimm S., Stanger B.Z., Leder P. (1996). RIP and FADD: Two “death domain”-containing proteins can induce apoptosis by convergent, but dissociable, pathways. Proc. Natl. Acad. Sci. USA.

[60] Shariat S.F., Desai S., Song W., Khan T., Zhao J., Nguyen C., Foster B.A., Greenberg N., Spencer D.M., Slawin K.M. (2001). Adenovirus-mediated transfer of inducible caspases: A novel “death switch” gene therapeutic approach to prostate cancer. Cancer Res..

[61] Yang X., Chang H.Y., Baltimore D. (1998). Autoproteolytic activation of pro-caspases by oligomerization. Mol. Cell.

[62] Shah V.R., Koster M.I., Roop D.R., Spencer D.M., Wei L., Li Q., Schwartz R.J., Chang J. (2007). Double-inducible gene activation system for caspase 3 and 9 in epidermis. Genesis.

[63] Chang D.W., Xing Z., Capacio V.L., Peter M.E., Yang X. (2003). Interdimer processing mechanism of procaspase-8 activation. EMBO J..

[64] Oberst A., Pop C., Tremblay A.G., Blais V., Denault J.B., Salvesen G.S., Green D.R. (2010). Inducible dimerization and inducible cleavage reveal a requirement for both processes in caspase-8 activation. J. Biol. Chem..

[65] Khaleghi S., Rahbarizadeh F., Ahmadvand D., Rasaee M.J., Pognonec P. (2012). A caspase 8-based suicide switch induces apoptosis in nanobody-directed chimeric receptor expressing T-cells. Int. J. Hematol..

[66] Carlotti F., Zaldumbide A., Martin P., Boulukos K.E., Hoeben R.C., Pognonec P. (2005). Development of an inducible suicide gene system based on human caspase 8. Cancer Gene Ther..

[67] Pajvani U.B., Trujillo M.E., Combs T.P., Iyengar P., Jelicks L., Roth K.A., Kitsis R.N., Scherer P.E. (2005). Fat apoptosis through targeted activation of caspase 8: A new mouse model of inducible and reversible lipoatrophy. Nat. Med..

[68] MacCorkle R.A., Freeman K.W., Spencer D.M. (1998). Synthetic activation of caspases: Artificial death switches. Proc. Natl. Acad. Sci. USA.

[69] Straathof K.C., Pule M.A., Yotnda P., Dotti G., Vanin E.F., Brenner M.K., Heslop H.E., Spencer D.M., Rooney C.M. (2005). An inducible caspase 9 safety switch for T-cell therapy. Blood.

[70] Gross A., Jockel J., Wei M.C., Korsmeyer S.J. (1998). Enforced dimerization of Bax results in its translocation, mitochondrial dysfunction and apoptosis. EMBO J..

[71] Lowe S.L., Rubinchik S., Honda T., McDonnell T.J., Dong J.Y., Norris J.S. (2001). Prostate-specific expression of Bax delivered by an adenoviral vector induces apoptosis in lncap prostate cancer cells. Gene Ther..

[72] Toyota H., Kondo S., Kyo S., Mizuguchi J. (2006). Enforced expression of a truncated form of Bax-α (TBax) driven by human telomerase reverse transcriptase (hTERT) promoter sensitizes tumor cells to chemotherapeutic agents or tumor necrosis factor-related apoptosis-inducing ligand (TRAIL). Anticancer Res..

[73] Chang E.C., Liu H., West J.A., Zhou X., Dakhova O., Wheeler D.A., Heslop H.E., Brenner M.K., Dotti G. (2016). Clonal dynamics in vivo of virus integration sites of T-cells expressing a safety switch. Mol. Ther. J. Am. Soc. Gene Ther..

[74] Barese C.N., Felizardo T.C., Sellers S.E., Keyvanfar K., di Stasi A., Metzger M.E., Krouse A.E., Donahue R.E., Spencer D.M., Dunbar C.E. (2015). Regulated apoptosis of genetically modified hematopoietic stem and progenitor cells via an inducible caspase-9 suicide gene in rhesus macaques. Stem Cells.

[75] Traversari C., Marktel S., Magnani Z., Mangia P., Russo V., Ciceri F., Bonini C., Bordignon C. (2007). The potential immunogenicity of the TK suicide gene does not prevent full clinical benefit associated with the use of TK-transduced donor lymphocytes in hsct for hematologic malignancies. Blood.

[76] Garin M.I., Garrett E., Tiberghien P., Apperley J.F., Chalmers D., Melo J.V., Ferrand C. (2001). Molecular mechanism for ganciclovir resistance in human T lymphocytes transduced with retroviral vectors carrying the herpes simplex virus thymidine kinase gene. Blood.

[77] Ciceri F., Bonini C., Stanghellini M.T., Bondanza A., Traversari C., Salomoni M., Turchetto L., Colombi S., Bernardi M., Peccatori J. (2009). Infusion of suicide-gene-engineered donor lymphocytes after family haploidentical haemopoietic stem-cell transplantation for leukaemia (the TK007 trial): A non-randomised phase I–II study. Lancet Oncol..

[78] Bondanza A., Hambach L., Aghai Z., Nijmeijer B., Kaneko S., Mastaglio S., Radrizzani M., Fleischhauer K., Ciceri F., Bordignon C. (2011). Il-7 receptor expression identifies suicide gene-modified allospecific CD8^+^ T-cells capable of self-renewal and differentiation into antileukemia effectors. Blood.

[79] Vago L., Oliveira G., Bondanza A., Noviello M., Soldati C., Ghio D., Brigida I., Greco R., Lupo Stanghellini M.T., Peccatori J. (2012). T-cell suicide gene therapy prompts thymic renewal in adults after hematopoietic stem cell transplantation. Blood.

[80] Vanden Berghe T., Linkermann A., Jouan-Lanhouet S., Walczak H., Vandenabeele P. (2014). Regulated necrosis: The expanding network of non-apoptotic cell death pathways. Nat. Rev. Mol. Cell Biol..

[81] Zhang D.W., Shao J., Lin J., Zhang N., Lu B.J., Lin S.C., Dong M.Q., Han J. (2009). RIP3, an energy metabolism regulator that switches TNF-induced cell death from apoptosis to necrosis. Science.

[82] Cho Y.S., Challa S., Moquin D., Genga R., Ray T.D., Guildford M., Chan F.K. (2009). Phosphorylation-driven assembly of the RIP1–RIP3 complex regulates programmed necrosis and virus-induced inflammation. Cell.

[83] Moquin D.M., McQuade T., Chan F.K. (2013). CYLD deubiquitinates RIP1 in the TNFα-induced necrosome to facilitate kinase activation and programmed necrosis. PLoS ONE.

[84] Wilson N.S., Dixit V., Ashkenazi A. (2009). Death receptor signal transducers: Nodes of coordination in immune signaling networks. Nat. Immunol..

[85] Li J., McQuade T., Siemer A.B., Napetschnig J., Moriwaki K., Hsiao Y.S., Damko E., Moquin D., Walz T., McDermott A. (2012). The RIP1/RIP3 necrosome forms a functional amyloid signaling complex required for programmed necrosis. Cell.

[86] Zhao J., Jitkaew S., Cai Z., Choksi S., Li Q., Luo J., Liu Z.G. (2012). Mixed lineage kinase domain-like is a key receptor interacting protein 3 downstream component of TNF-induced necrosis. Proc. Natl. Acad. Sci. USA.

[87] Cai Z., Jitkaew S., Zhao J., Chiang H.C., Choksi S., Liu J., Ward Y., Wu L.G., Liu Z.G. (2014). Plasma membrane translocation of trimerized MLKl protein is required for TNF-induced necroptosis. Nat. Cell Biol..

[88] Linkermann A., Green D.R. (2014). Necroptosis. N. Engl. J. Med..

[89] Borst P., Rottenberg S. (2004). Cancer cell death by programmed necrosis?. Drug Resist. Updates Rev. Comment. Antimicrob. Anticancer Chemother..

[90] Moriwaki K., Chan F.K. (2013). RIP3: A molecular switch for necrosis and inflammation. Genes Dev..

[91] Orozco S., Yatim N., Werner M.R., Tran H., Gunja S.Y., Tait S.W., Albert M.L., Green D.R., Oberst A. (2014). RIPK1 both positively and negatively regulates RIPK3 oligomerization and necroptosis. Cell Death Differ..

[92] Wu X.N., Yang Z.H., Wang X.K., Zhang Y., Wan H., Song Y., Chen X., Shao J., Han J. (2014). Distinct roles of RIP1-RIP3 hetero- and RIP3-RIP3 homo-interaction in mediating necroptosis. Cell Death Differ..

[93] Tanzer M.C., Matti I., Hildebrand J.M., Young S.N., Wardak A., Tripaydonis A., Petrie E.J., Mildenhall A.L., Vaux D.L., Vince J.E. (2016). Evolutionary divergence of the necroptosis effector MLKL. Cell Death Differ..

[94] Minagawa K., Jamil M.O., Al-Obaidi M., Pereboeva L., Salzman D., Erba H.P., Lamb L.S., Bhatia R., Mineishi S., di Stasi A. (2016). In vitro pre-clinical validation of suicide gene modified anti-CD33 redirected chimeric antigen receptor T-cells for acute myeloid leukemia. PLoS ONE.

[95] Diaconu I., Ballard B., Zhang M., Chen Y., West J., Dotti G., Savoldo B. (2017). Inducible caspase-9 selectively modulates the toxicities of CD19-specific chimeric antigen receptor-modified T-cells. Mol. Ther. J. Am. Soc. Gene Ther..

[96] Minagawa K., Zhou X., Mineishi S., di Stasi A. (2015). Seatbelts in car therapy: How safe are cars?. Pharmaceuticals.

[97] Ando M., Nishimura T., Yamazaki S., Yamaguchi T., Kawana-Tachikawa A., Hayama T., Nakauchi Y., Ando J., Ota Y., Takahashi S. (2015). A safeguard system for induced pluripotent stem cell-derived rejuvenated T-cell therapy. Stem Cell Rep..

[98] Cheng F., Ke Q., Chen F., Cai B., Gao Y., Ye C., Wang D., Zhang L., Lahn B.T., Li W. (2012). Protecting against wayward human induced pluripotent stem cells with a suicide gene. Biomaterials.

[99] Ramos C.A., Asgari Z., Liu E., Yvon E., Heslop H.E., Rooney C.M., Brenner M.K., Dotti G. (2010). An inducible caspase 9 suicide gene to improve the safety of mesenchymal stromal cell therapies. Stem cells.

[100] Amariglio N., Hirshberg A., Scheithauer B.W., Cohen Y., Loewenthal R., Trakhtenbrot L., Paz N., Koren-Michowitz M., Waldman D., Leider-Trejo L. (2009). Donor-derived brain tumor following neural stem cell transplantation in an ataxia telangiectasia patient. PLoS Med..

[101] Pegram H.J., Park J.H., Brentjens R.J. (2014). CD28z cars and armored cars. Cancer J..

[102] Hacein-Bey-Abina S., Hauer J., Lim A., Picard C., Wang G.P., Berry C.C., Martinache C., Rieux-Laucat F., Latour S., Belohradsky B.H. (2010). Efficacy of gene therapy for X-linked severe combined immunodeficiency. N. Engl. J. Med..

[103] Hacein-Bey-Abina S., Garrigue A., Wang G.P., Soulier J., Lim A., Morillon E., Clappier E., Caccavelli L., Delabesse E., Beldjord K. (2008). Insertional oncogenesis in 4 patients after retrovirus-mediated gene therapy of SCID-X1. J. Clin. Investig..

[104] Boztug K., Schmidt M., Schwarzer A., Banerjee P.P., Diez I.A., Dewey R.A., Bohm M., Nowrouzi A., Ball C.R., Glimm H. (2010). Stem-cell gene therapy for the wiskott-aldrich syndrome. New Engl. J. Med..

[105] Cavazzana-Calvo M., Payen E., Negre O., Wang G., Hehir K., Fusil F., Down J., Denaro M., Brady T., Westerman K. (2010). Transfusion independence and HMGA2 activation after gene therapy of human β-thalassaemia. Nature.

[106] Braun C.J., Boztug K., Paruzynski A., Witzel M., Schwarzer A., Rothe M., Modlich U., Beier R., Gohring G., Steinemann D. (2014). Gene therapy for wiskott-aldrich syndrome—Long-term efficacy and genotoxicity. Sci. Transl. Med..

[107] Morgan R.A., Yang J.C., Kitano M., Dudley M.E., Laurencot C.M., Rosenberg S.A. (2010). Case report of a serious adverse event following the administration of T-cells transduced with a chimeric antigen receptor recognizing ERBB2. Mol. Ther. J. Am. Soc. Gene Ther..

[108] Thirabanjasak D., Tantiwongse K., Thorner P.S. (2010). Angiomyeloproliferative lesions following autologous stem cell therapy. J. Am. Soc. Nephrol..

[109] Marks P.W., Witten C.M., Califf R.M. (2017). Clarifying stem-cell therapy’s benefits and risks. N. Engl. J. Med..

[110] Uchida N., Evans M.E., Hsieh M.M., Bonifacino A.C., Krouse A.E., Metzger M.E., Sellers S.E., Dunbar C.E., Donahue R.E., Tisdale J.F. (2013). Integration-specific in vitro evaluation of lentivirally transduced rhesus CD34^+^ cells correlates with in vivo vector copy number. Mol. Ther. Nucleic Acids.

[111] Pandey M.K., Prasad S., Tyagi A.K., Deb L., Huang J., Karelia D.N., Amin S.G., Aggarwal B.B. (2016). Targeting cell survival proteins for cancer cell death. Pharmaceuticals.

